# Blood Stains

**Published:** 1872-08

**Authors:** Henry Haacke

**Affiliations:** From Caspar’s Gerichtliche Medicin, Berlin


*Blood Stains.* By HENRY HAACKE. From Caspar's Gerichtliche Medicin, Berlin. 1871.

In criminal trials it is often necessary to determine whether certain stains on weapons, furniture, doors, walls, vessels or clothing, resembling blood stains, are really such or not, or if a knife or other instrument, showing no blood stains, could nevertheless have been used in causing a certain wound. It is claimed, perhaps, for the defense, that the accused could not possibly have inflicted the wound with the knife, because it shows no blood stains; or, the existence of blood stains being admitted, it is contended that they were caused by the blood of some animal. The difficulty and importance of the question, and its frequent occurrence in courts of law, led to continued searches after methods to recognize blood; but not until quite recently has it been possible to reach any certainty in differential diagnosis in doubtful cases of blood stains, and the numerous former methods of examination,* all more or

* Compare: Orfila Traite de Med. leg. 2nd Ed. II. p. 564. Lassaigne, Revue Medic., August, 1821. Barruele, Annales d'Hygiene publique, 1829. Chevalier, in Poggendorf's Annalen, 1838, No. 9. Barruel and Lesneur, Archives de Med., 1833, 1-2 Serie. H. Rose, in Casper's Vierteljahrschr, 1853, iv. p. 295. C. Schmidt, die Diagnostik verdachtiger Flecke in Criminalfallen Mittan u. Leipless complicated and uncertain, have been supplanted by simpler and more reliable means of recognition. But we must first state what has not been mentioned anywhere else, that *weapons may have been used in causing wounds, although even the closest scrutiny fails to detect the slightest trace of blood on them.*

The following is a case in point:

The body of a young man was found in the *Thiergarten*, near Berlin, with a large cut in the throat. By its side lay the ruptured noose of a small rope; also a knife, without the slightest blood stain. Neither the hands nor any other part of the body showed a trace of blood, though a large quantity of blood which had flowed from the wound, was found on the ground. The linen, and especially the shirt of the deceased, was clean. From these circumstances, and from the fact that the earth in a circuit of nearly two yards around showed evidence of a struggle, the police inferred that a murder had been committed. A closer examination revealed marks of strangulation. The cut separated the pomum Adami from the os hyoides; it was four inches in length, half an inch in width, had sharp, bloody margins, and had on the right, laid open the trachea; ending on the left, in an acute angle, barely passing through the epidermis. On the left side of the neck there were several superficial semi-lunar abrasions, cicatrized, but without suggillation; another abrasion in the same place, was somewhat deeper, showing a loss of substance of the skin almost as large as a lentil. There were, besides, several slight abrasions both on the lower jaw and on the right side of the neck near the wound. Under the chin, to the right, there was a small spot, denuded from the cuticle, and dried up; without suggillation. The lids of both eyes were very red. A close inspection showed numerous capillary ecchymoses of the size of the point of a pin, so that the skin of the lids presented the appearance of being dotted all over with fine red points. The conjunctivæ were slightly red and showed a few ecchymoses. On the forehead there were numerous capillary ecchymoses of the size of the point of a pin. There zig, 1848. B. Rittir, uber die Ermittelung von Blut., Samen und Excrementen-flecken in Criminalfallen Ein gekronte Preisschrift 2. Aufl. Wurzburg, 1854 (mit reicher Literatur), Lassaigne, Annales d'Hygiene publique, 1857.

More recent works: B. Ritter (Geschichtliche Darstellung aller methoden), in Henke's Zeitschrift, 1860, iii. Pfaff, Anleitung zur Vornahme gerichtsarztlicher Blutuntersuchungen 2. Aufl. Plauen, 1863, Zwei Abhandlungen von Erpenbeck und Pfaff in Vierteljahrsschrift, 1862, xxi. 1. u. 2. Heft. Hoppe-Seyler, Handbuch der chemischen Analyse, Berlin, 1865. Sonnenschein, Handbuch der gerichtlichen chemie, 1869. Herapath, on the use of the spectroscope and microspectroscope in the discovery of blood stains and dissolved blood. Brit. Med. Leg. Journal, 1866. Blondlot, de la Constation med. leg. des taches de sang par la formation des cristaux d'hemine, Ann. d'hygiene publique, 1868. Taylor, on the Guaiac process for the detection of blood in med. leg. cases, Guy's Hospital Rep., 1868, xiv. Falk, zur spectroscopie in d. forens. Praxis. Vierteljarsschrift f. gerichtl. med. 1867, S. 234.

was a blood stain on the dorsal surface of the left thumb, and a similar one on the palm of the left hand. No spermatozoa in the urethra.

On dissection, it appeared that the carotis and vena jugularis were uninjured. There was a small opening in the vena facialis, branching off from the jugularis. The ligam, thyreo-hyoideum had been divided, as also the posterior wall of the pharynx. Around the wound, especially at both ends, appeared infiltrations of blood in the cellular tissue. In the trachea and larynx there was frothy blood, an abundance of which was found in the large and smaller bronchi. The mucous membranes of the trachea and larynx were much congested, and under the mucous membrane of the larynx there were numerous light-red ecchymoses of the size of a lentil. The lungs well expanded; the superior portion of the anterior lobe light greyish-red, anæmic, dry; but the inferior portion of the posterior lobe hyperæmic, of a dark bluish-red color. Incisions produced distinct red spots of the size of a pea on the anterior lobes, several groups of lung-cells being filled with blood. Heart normal, containing a good deal of dark liquid blood, but no coagulated blood. The organs of the abdomen contained but little blood, but the vena cava was full of dark liquid blood.

The knife was a very large one, with a long, rough handle. The blade convex, three inches long, in its greatest width half an inch. The most careful examination by Professors Sonnerschein and Caspar showed no blood stains on the knife. Death had, therefore, ensued not from excessive hemorrhage, but from suffocation caused by the blood from the injured vessel flowing and being breathed into the trachea.

The marks of strangulation had been caused in life, and had preceded the cut in the throat. This would seem to follow from the condition of the eyelids, the ecchymoses of the conjunctivæ, and from the fact that the rope was found near the body. If this be so, the marks of strangulation must be explained by an unsuccessful attempt at suicide. Had another person effected strangulation before the cut, the deceased would have died from suffocation it is true, but not from suffocation in his own blood, as was here the case. A stranger might have applied the rope after death, in order to simulate suicide after the infliction of the cut, but in that case the rope could not have produced the effect described, and it would not have been found *near* the body, but around its neck.

The absence of all traces of resistance is also against the assumption of homicide.

The numerous abrasions on the throat are remarkable; but they must be ascribed in this case (as in many similar cases) to the finger nails of the deceased, who hastily applied and tore off the rope. The cut in the throat has been inflicted with the left hand. This follows from the presence of blood on the latter, as also from the situation and direction of the wound. The scanty traces of blood on the hand and their absence on the knife are explained by the great size of the knife, the length of the handle and the smallness of the injury of the vessel. Hemorrhage did not commence until both knife and hand had been taken away from the region of the throat. The conclusions arrived at were confirmed by further developments.

EXAMINATION OF BLOOD.

It is, of course, frequently possible to recognize blood on weapons and other objects by simple inspection with the naked eye. I will merely remark here, that blood stains present various shades of color, as dark-brown red, brown and brown-green, olive-light green, light red, and no color at all, according as they have dried rapidly or slowly, by great heat or otherwise, or were washed when still moist or already dry; so that nobody would recognize some of them as blood stains, but rather as mucus, pus, urine or anything else.

A closer inspection alone can decide in doubtful cases.

If the blood is still fresh, it will not be difficult to recognize, by means of the microscope, the red corpuscles of the blood, whose form and nature are supposed to be well known. In the fresh state, it is also possible to measure them and distinguish them, by their diameter, from those of other animals.

According to Schmidt the diameters of blood cells are as follows:

| | | | |
|---|---|---|---|
| Of man | 0.0077 | Mm. | [0.0074—0.0080]. |
| " dogs | 0.0070 | " | [0.0066—0.0074]. |
| " rabbits | 0.0064 | " | [0.0060—0.0070]. |
| " rats | 0.0064 | " | [0.0060—0.0068]. |
| " hogs | 0.0062 | " | [0.0060—0.0c65]. |
| " mice | 0.0061 | " | [0.0058—0.0065]. |
| " oxen | 0.0058 | " | [0.0054—0.0060]. |
| " cats | 0.0056 | " | [0.0053—0.0060]. |
| " horses | 0.0057 | " | [0.0053—0.0060]. |
| " sheep | 0.0045 | " | [0.0040—0,0048]. |

On an average of twenty measurements the blood cells of the chicken are 0.0076 Mm. in width and 0.0027 Mm. in length. The white blood cells of warm-blooded animals are larger than the red.

It is well known that blood cells swell by the action of water, whilst the abstraction of water and drying up, gives them a ragged appearance. Dried blood cells look somewhat like the pavement of a street which is cracked here and there (Sonnenschein.)

In the case of such dried up blood the measurements become uncertain.

But even in dried up blood we succeed in producing its elementary forms. For this purpose we treat the blood with absolute alcohol of 95 per cent., let it dry and repeat the experiment several times, and then examine it with the addition of a liquid consisting of equal parts of ether and amyl alcohol (Gwosdew),* a solution of chloride of sodium or even distilled water. Separate blood cells may be obtained by treating the blood with a brush in a watch glass, by drying, scraping and final examination with the addition of a liquid. Still, even blood cells lying together in heaps may often be very well recognized as such.

A second method of examination is that by the spectroscope. For this purpose the blood is sufficiently diluted, which is best done, according to Helwig and Falk, by a solution of iodide of potassium (1:4). If blood is really present, the bands of absorption of the hæmatin will appear between D and E of Frauenhofer's spectrum. Blood is, thirdly, demonstrated with certainty by the production of the crystals of hæmin. Teichman† discovered that by the effect of acetic acid on blood, crystals of hæmin are formed. The further experiments of Erdmann, Buchner, Simon and others, and, later, of Helwig, Gwosdew and Falk have simplified this method to such a degree as to render it, as well as the two methods previously described, available for forensic purposes. The bloody objects are treated in a test tube with glacial acetic acid; a few small grains of chloride of sodium are added, and after boiling the liquid, it is evaporated in a watch glass on a hot stove in the sandbath or in the sun. If liquids are to be examined for blood, it is better to previously evaporate them. Caspar says he examines, generally, according to the still simpler method of Erdmann‡ by adding to the stain, previously removed as completely as possible from the object, besides a small grain of chloride of sodium, glacial acetic acid, in drops, heating cautiously and then allowing it to cool. This examination may well follow that for blood corpuscles according to the method above described. Falk recommends, especially in stains on dark articles of clothing, but also in other cases, to first extract the blood by means of a solution of iodide of potassium and then to examine the solution for hæmin. This method makes the addition of chloride of sodium superfluous. The crystals of hæmin are rhombic or rhomboidal in shape, of different sizes and varying in color, slightly yellow, yellow, yellow-red, blood-red or brown-red. They are frequently grouped in the form of a cross or star. In very small quantities of blood they

* Sitzungsbericht der Kais, Academie d. Wissensch. Bd. liii, 1866.

† Teichman, uber die Crystallisation der organ. Bestandtheile des Blutes, in Henle u. Pfeiffer's Zeitschr. f. rationelle med. iii. 3. S. 371.

‡ Journ. f. Pract. Chemie Bd. 85, p. 1.

are often so thin as to have almost no color at all. Good representations are found in Funke's atlas of physiological chemistry. As the crystals may be mistaken for dirt, and, especially, for crystals of purpurate of ammonia, we recommend to examine them with the polariscope. Rollett * has shown that the crystals change their color four times, but so that dark and light alternate, if a *nickel* prisma is placed between the eye and the polarizer and the prisma is turned 360°.

The ozonizing power of hæmatin may, finally, be used for the diagnosis of blood, if hæmatin is added to tincture of guaiac mixed with ether or other matter containing ozone. This is so delicate a test as to be effective even in cases where the blood stains have been washed out until nothing can be distinguished by the naked eye. Its employment is most appropriate when all the circumstances of the case are in favor of assuming an absence of blood.

All these methods are insufficient to distinguish human blood from animal blood.

Caspar says it appears from his own experiments and those of Chevalier, that the so-called discovery of Barruel, to distinguish human blood from animal blood by the odor, must be rejected as unsafe and unfit for forensic purposes. The same may be said of Neumann's method to distinguish the blood of the different animals by the microscopic formations produced through evaporation at a temperature of 10° to 12° R.

Experiments to distinguish menstrual blood from blood caused by wounds, have hitherto been unsuccessful. Nor has it been possible to determine the age of blood stains with much degree of certainty.

CONCLUDING REMARKS.

1. *Weapons.* If the weapons are of shining metals, such as the tools of mechanics, it will be difficult to mistake fresh blood stains, though dried, and examined with the naked eye only, for other stains. Blood stains are of a light red, if only a thin layer of blood adheres to the weapon, and of a dark red if more blood is present. If light, especially sunlight, falls on them, they become irisated. They may be distinguished from rust by heating the metal, when the blood comes off in scales, but the rust undergoes no change. In addition, it will be necessary to submit the substance, after scraping it off, to the tests before described.

It is impossible to distinguish, with the naked eye, old dry blood stains from rust stains. They differ in the following particulars: 1. Rust stains are lighter and duller; blood stains darker and

* Rollett, uber den Pleochroismus der Hamin crystale nebst einer kurzen anleitung zur Untersuchung desselben. Wiener Med. Wochenschr., 1862, No. 23.

more brilliant (irisated). 2. Rust stains adhere better and remain on heating; blood stains come off in scales. 3. Rust dissolves easily in muriatic acid; blood stains do not. 4. Detached blood stains may be melted with sodium, and the product may, on cooling, be examined for cyanogen. 5. A blood stain which has become insoluble in water, from oxide of iron (forming rust), may be dissolved in potassa, and the solution may then be tested. It is necessary, however, to remember that iron rust generally contains ammonia.

2. *Fresh blood stains on furniture, doors, wall paper, wooden handles, boots, etc.*, of a dark color, that cannot be distinguished in daylight, become visible by the use of artificial light, as for instance that of a wax candle (Ollivier and Pillon). The stains must be scraped off from these articles and examined as before directed.

3. *Blood stains on clothing or linen* may either be scraped off or extracted, or they may be examined in their original state, according to the methods described. The chemical methods recommended by Rose,* Morin,† Wiehr,‡ Bryk,§ and others, are less certain. Rust stains on linen cannot be mistaken for blood stains, even when washed. Only fresh stains of acetate or sesqui-oxide of iron may be mistaken for fresh blood stains, but they retain their red color, whilst blood stains soon become darker or brownish-red. All other preparations of iron, and also the one just named, when washed, become yellow by being changed into hydrated sesquioxide of iron. These stains of iron, when washed, retain much more definite outlines than blood stains; and when the outlines of iron stains disappear, they represent only different shades of yellow, whilst blood shows reddish or greenish shades. They differ also in the following particulars: 1. An iron stain, boiled with diluted muriatic acid, forms, immediately, a yellowish solution; but a blood stain undergoes no change. 2. An iron stain turns dark blue (Prussian blue) on being acidulated by a few drops of diluted muriatic acid, with the addition of ferro-cyanide of potassium; a blood stain does not. 3. An iron stain, by diluted muriatic acid, in a moderate heat, may be obliterated until there is no reaction even to ferro-cyanide of potassium nor to the resin of guaiacum; whilst a blood stain, even when washed until its color disappears, shows this reaction, on being treated in the same way with muriatic acid. So, too, pus, urine and fæces do not show this reaction with the resin of guaiacum.—*The Clinic.*

* Caspar, Vierteljahrsschrift, IV, S. 255.

† Archiv. der Pharmacie, Hft 2, '64, S. 192.

§ Wien, med. Wochenschrift, 1858, S. 789, and the former editions of this work.

